# ABA-Induced Vegetative Diaspore Formation in *Physcomitrella patens*

**DOI:** 10.3389/fpls.2019.00315

**Published:** 2019-03-19

**Authors:** M. Asif Arif, Manuel Hiss, Marta Tomek, Hauke Busch, Rabea Meyberg, Stefanie Tintelnot, Ralf Reski, Stefan A. Rensing, Wolfgang Frank

**Affiliations:** ^1^Plant Molecular Cell Biology, Department Biology I, Ludwig-Maximilians-Universität München, LMU Biocenter, Planegg-Martinsried, Germany; ^2^Plant Cell Biology, Faculty of Biology, University of Marburg, Marburg, Germany; ^3^Plant Biotechnology, Faculty of Biology, University of Freiburg, Freiburg, Germany; ^4^Lübeck Institute of Experimental Dermatology, University of Lübeck, Lübeck, Germany; ^5^Signalling Research Centres BIOSS and CIBSS, University of Freiburg, Freiburg, Germany; ^6^BIOSS Centre for Biological Signalling Studies, University of Freiburg, Freiburg, Germany

**Keywords:** moss, ABA, diaspore, brachycyte, gene expression, cell wall

## Abstract

The phytohormone abscisic acid (ABA) is a pivotal regulator of gene expression in response to various environmental stresses such as desiccation, salt and cold causing major changes in plant development and physiology. Here we show that in the moss *Physcomitrella patens* exogenous application of ABA triggers the formation of vegetative diaspores (brachycytes or brood cells) that enable plant survival in unfavorable environmental conditions. Such diaspores are round-shaped cells characterized by the loss of the central vacuole, due to an increased starch and lipid storage preparing these cells for growth upon suitable environmental conditions. To gain insights into the gene regulation underlying these developmental and physiological changes, we analyzed early transcriptome changes after 30, 60, and 180 min of ABA application and identified 1,030 differentially expressed genes. Among these, several groups can be linked to specific morphological and physiological changes during diaspore formation, such as genes involved in cell wall modifications. Furthermore, almost all members of ABA-dependent signaling and regulation were transcriptionally induced. Network analysis of transcription-associated genes revealed a large overlap of our study with ABA-dependent regulation in response to dehydration, cold stress, and UV-B light, indicating a fundamental function of ABA in diverse stress responses in moss. We also studied the evolutionary conservation of ABA-dependent regulation between moss and the seed plant *Arabidopsis thaliana* pointing to an early evolution of ABA-mediated stress adaptation during the conquest of the terrestrial habitat by plants.

## Introduction

The moss *Physcomitrella patens* is a model plant for studies on evolutionary developmental (evo-devo) processes, molecular responses and abiotic stress adaptation. The relevant features include a fully sequenced genome, a unique evolutionary position approximately half way between green algae and angiosperms, very efficient gene-targeting by homologous recombination and a haploid-dominant life cycle that enables direct analysis of mutants without the need for time consuming back crosses (Schaefer and Zryd, 1997; Hofmann et al., 1999; Kamisugi et al., 2005; Rensing et al., 2008). The development of the comparatively few tissue types is controlled by plant hormones. *P. patens* is a poikilohydric species whose water potential equilibrates quickly with that of the environment, a feature that was lost during seed plant evolution. *P. patens* is highly tolerant against UV-B, salt, drought and osmotic stresses (Frank et al., 2005b; Wolf et al., 2010) and several studies have been performed to unravel the molecular mechanisms underlying this broad abiotic stress tolerance (Frank et al., 2005b, 2007; Saavedra et al., 2006; Cuming et al., 2007; Qudeimat et al., 2008; Wang X. et al., 2009; Wang X.Q. et al., 2009; Richardt et al., 2010; Wolf et al., 2010; Komatsu et al., 2013; Beike et al., 2015; Khraiwesh et al., 2015).

The phytohormone abscisic acid (ABA) is a central mediator of various abiotic stress responses (Yamaguchi-Shinozaki and Shinozaki, 2006). The initial steps of ABA biosynthesis take place in plastids, starting with the methyl erythritol phosphate (MEP) pathway that leads to the production of carotenoids (Ruiz-Sola and Rodriguez-Concepcion, 2012). These are metabolized to zeaxanthin, which in turn is converted to violaxanthin and subsequently to *trans*-neoxanthin. The stress-induced 9-*cis*-epoxycarotenoid dioxygenase (NCED) catalyzes the final rate limiting steps of xanthoxin production that leaves the plastid and gives rise to ABA (Frey et al., 2012; Behnam et al., 2013; Finkelstein, 2013; Espasandin et al., 2014). Endogenous ABA levels are also controlled by catabolism and inactivation via conjugation to other molecules with the most common conjugate being the glucosyl ester synthesized by glucosyltransferases (Lim et al., 2005).

The response to ABA is initiated by a signaling pathway, which includes the activation of kinase cascades. ABA binds to a family of receptor proteins [PYRABACTIN RESISTANCE1 (PYR1)/PYR1-LIKE (PYL)/REGULATORY COMPONENTS OF ABA RECEPTORS (RCAR)] and promotes complex formation between PYR/PYL/RCAR proteins and phosphatase 2Cs (PP2Cs). In the absence of ABA, SnRK2 (sucrose non-fermenting 1-related protein kinase 2) protein kinases are kept dephosphorylated whereas in the presence of ABA, ABA-bound receptors sequester PP2Cs allowing the phosphorylation of SnRK2 and subsequent activation of ABA-responsive element binding proteins/factors (AREBs/ABFs) by phosphorylation (Kagaya et al., 2002; Furihata et al., 2006; Xie et al., 2012). Promoters of ABA-responsive genes possess ABA-responsive elements (ABREs) that can be bound by ABRE-BINDING PROTEINS (ABREBPs) and confer transcriptional activation upon an ABA stimulus (Busk and Pages, 1998; Narusaka et al., 2003). In addition, several transcription factors (TFs) of the MYB, MYC, NAC, bZip, WRKY, and DREB protein families regulate gene expression in an ABA-dependent manner (Abe et al., 2003; Fujita et al., 2004; Lee et al., 2010). *P. patens* ABREs have been described (Timmerhaus et al., 2011) and they act together with the above mentioned TF families to convey ABA responses (Qudeimat et al., 2008; Richardt et al., 2010). In addition to transcriptional regulation, ABA signaling also targets membrane components, proton pumps and ion channels (Zhang et al., 2004; Demir et al., 2013; Rodriguez et al., 2014; Lind et al., 2015). In seed plants and in bryophytes ABA protects against adverse environmental conditions and the contribution of ABA to abiotic stress responses has been particularly studied in seed plants. Important responses triggered by ABA include stomatal closure, maintenance of water balance, regulation of ion channels, stress signaling, changes in gene expression, promoting senescence, seed dormancy, and development (Zhang et al., 1987; Macrobbie, 1997; Busk and Pages, 1998; Leckie et al., 1998; Finkelstein, 2013; Takasaki et al., 2015; Suzuki et al., 2016; Kumar et al., 2017).

The core components of ABA signaling and the ABA response are conserved between *P. patens* to angiosperms including PYL, ABI1 and ABI2 (PP2C proteins), ABI3 (B3-domain containing transcription factor), ABI4 (AP2-type transcription factor), and OST1 (SnRK2) (Komatsu et al., 2009, 2013; Khandelwal et al., 2010; Sakata et al., 2014; Saruhashi et al., 2015; Stevenson et al., 2016). *P. patens* acquires an increased freezing, hyperosmosis and dehydration tolerance by ABA treatment (Machuka et al., 1999; Kamisugi and Cuming, 2005; Marella et al., 2006; Nagao et al., 2006; Oldenhof et al., 2006; Wang et al., 2010; Bhyan et al., 2012; Amagai et al., 2018; Zhao et al., 2018). Furthermore, an increase in endogenous ABA levels is caused by osmotic stresses in *P. patens* (Minami et al., 2003). Exogenous ABA application also affects growth and differentiation in mosses and liverworts (Goode et al., 1993b; Schnepf and Reinhard, 1997; Takezawa et al., 2011). *P. patens* protonema tissue tolerates water loss up to 92% on a fresh weight basis, but cannot survive complete desiccation (Frank et al., 2005b). However, ABA pretreatment of protonema enables it to survive complete desiccation (Khandelwal et al., 2010; Koster et al., 2010).

In angiosperms, ABA is a positive regulator of seed dormancy and high ABA levels delay seed germination under unfavorable environmental conditions. In mosses ABA induces the development of spherical, thick-walled cells called brachycytes (brood cells) in the protonema tissue that serve as vegetative diaspores which are tolerant to desiccation and freezing (Goode et al., 1993b; Schnepf and Reinhard, 1997; Decker et al., 2006; Zhao et al., 2018). Although a few studies in *P. patens* have been performed addressing ABA-dependent transcriptional changes (Cuming et al., 2007; Richardt et al., 2010; Komatsu et al., 2013; Khraiwesh et al., 2015; Stevenson et al., 2016), no studies have been performed to investigate cell wall thickening and related morphological changes together with coupled molecular actions of ABA on *P. patens* in detail. Here, we carried out genome-wide gene expression profiling in response to ABA and define the early (30, 60, and 180 min) molecular response of *P. patens* to ABA. Moreover, we characterize cell wall thickening and vegetative diaspore formation upon ABA treatment and analyze crosstalk of transcriptional regulation between ABA and abiotic stressors.

## Materials and Methods

### Plant Material

The moss *P. patens* (Hedw.) Bruch & Schimp accession Gransden was used in this study. Plant material was axenically cultivated in liquid minimal medium (250 mg/L KH_2_PO_4_, 250 mg/L KCl, 250 mg/L MgSO_4_ 7H_2_O, 1,000 mg/L Ca(NO_3_)_2_ 4H_2_O, and 12.5 mg/L FeSO_4_ 7H_2_O) under standard growth conditions as described previously (Frank et al., 2005a). After 4 days protonemal tissue was disrupted for 10 s with an Ultra-Turrax device (IKA, Staufen, Germany) and transferred into fresh liquid medium.

### ABA Application

(+)-*cis*, *trans*-abscisic acid (ABA; Duchefa) was added to freshly homogenized protonemal liquid cultures that were adjusted to 100 mg/L dry weight. ABA was dissolved in 100 μM KOH and mock treatments were performed with 100 μM KOH only.

### Microscopy

Light and epifluorescence microscopy was performed either with an Olympus BX41 microscope or with a Zeiss Axioplan and images were taken with a Canon EOS D30 camera. Confocal microscopy was performed with a Zeiss LSM 510 UV microscope, excitation 543 nm; emission spectra for chlorophyll and propidium iodide were separated by linear unmixing. For cell wall staining, propidium iodide (Fluka) was added to a final concentration of 4% and slides were washed with medium after 5 min incubation.

Cryo-SEM analysis was performed using a Philips XL30 ESEM with Cryo Preparation Unit Gatan Alto 2500. Plant material was applied to a specimen holder with freeze hardening glue and biological samples were preserved by fast-freezing in liquid nitrogen. Afterward the specimen holder was inserted into the sputter chamber and coated with gold.

For TEM, after removal of supernatant, 2.5% glutaraldehyde in 50 mM cacodylate buffer (pH 5.7) was added to protonemal culture and incubated for 5 h. Samples were washed five times for 10 min with cacodylate buffer, embedded in low melting grade agarose (Sigma) and fixed with 1% OsO_4_ for 4 h at 4°C. After washing with cacodylate and 2× water, samples were dehydrated using an ethanol series and finally washed two times each with ethanol and xylol and imbibed in xylene-epoxy resin 1:1 overnight. Samples were embedded in Epon 812 resin (Sigma) by incubation for 2 days at 60°C. Ultrathin slices (70–100 μm) were cut with a Leica microtome and observed in an electron microscope CM 10 (Philips).

### Microarray Expression Profiling

RNA isolation, processing of RNA, microarray hybridization, washing and scanning as well as feature extraction and normalization were carried out as described previously (Wolf et al., 2010). Briefly, differentially expressed genes (DEGs) were called using Cyber-T (Baldi and Long, 2001) with false discovery rate (FDR) correction according to Benjamini and Hochberg (1995), using a *q*-value cutoff of 0.05. For comparison of different lists, Venn diagrams were created with the online tool Venny (Oliveros, 2007) while UpSet plots were generated as described previously (Khan and Mathelier, 2017).

### Expression Profiling by qRT-PCR

For cDNA synthesis, 4 μg total RNA was treated with DNase I (Fermentas, Sankt Leon-Rot, Germany) for 1 h at 37°C in order to eliminate remaining genomic DNA. The enzyme was inactivated by heating at 65°C for 10 min. Reverse transcription was performed according to the TaqMan Applied Biosystems (Roche) manufacturer’s recommendations. qRT-PCR reactions with 90 ng of cDNA were performed with the SensiMix^TM^ SYBR No-Rox Kit (Bioline) according to the manufacturer’s recommendations. All qRT-PCRs were performed in three biological and three technical replicates. The qRT-PCR program was adjusted to initial denaturation and hot start at 95°C for 10 min followed by 40 cycles of amplification with 96°C for 15 s, 60°C for 30 s, and 72°C for 20 s. The SYBR Green signals were measured at each cycle and melting curves were calculated to prove primer specificities. Gene expression values were normalized to the *PpEF1α* control gene and the relative quantifications were calculated based on Advance Relative Quantification provided by the LightCycler^®^ 480 software release 1.5.0. Quantifications were based on the delta-delta Ct method.

### Gene Ontology Analysis

The gene ontology (GO) enrichment analyses for *P. patens* and *A. thaliana* genes were performed with PANTHER (Mi et al., 2010) and the PANTHER database. PANTHER’s tool accesses a comprehensive list of GO annotations from the GO Consortium and is updated monthly to ensure the most current annotation data. To provide an overview on over-/under-represented GO terms Fisher’s exact test with subsequent Bonferroni correction was performed and *q*-values < 0.05 were considered as significant. For the GO clouds in Supplementary Figure S1, GO bias analyses were performed as in Widiez et al. (2014) based on the v1.6 GO association and visualized using Wordle^1^. For Supplementary Figure S3 we used the data from this study as well as from a previous study employing the same microarray (Hiss et al., 2014). Genes with highest expression in either protonema treated 180 min with ABA (250) or brown sporophytes (55) were identified, so that maximum expression of all replicates over the complete array dataset had to have a lower expression than the minimum expression of the ABA treated/brown sporophyte replicates.

### Global Transcriptome Analysis

Global transcriptome analysis was calculated as previously described (Busch et al., 2013). The rationale behind the analysis is the idea that cells change their transcriptomes after stimulation in concerted fashion, i.e., there are hundreds to thousands of genes that are up- or down-regulated together, either immediately or in delayed fashion. In order to detect those genes that considerably contribute to the concerted change of the transcriptome, we first ranked all genes according to their variance in expression after stimulation starting/ending with those genes that have the strongest/weakest response to the stimulation. We then grouped the ranked list of genes into subsets of 50–500 genes and compared the change in mutual information (MI) and Pearson correlation (PC) after stimulation of these subsets with the same change of the whole transcriptome. Gene subsets that considerably contribute to the overall change in gene expression should behave similar to the whole transcriptome, as quantified by a minimal difference of the MI and PC between the respective subsets and the transcriptome. Thus, we consider all genes that show the maximal temporal response as well as those that are most correlated with the overall transcriptome change as important for the ABA response, which is depicted in Figure 6. In brief, the method calculates the Euclidean distance of the PC and the MI between the whole transcriptome and subsets of genes that have been ordered from large to small temporal response. The idea is to determine those genes that are (i) strongly regulated over time or (ii) whose temporal dynamics correlate well with the whole transcriptome (computed between the global and ordered gene subset response for subsets of different sizes). In an alternative approach we calculated the significance of differential regulation over time for each gene by fitting a reduced model to all time points using a 3rd order polynomial or a full model by fitting the ABA response and control separately. The *p*-value was then calculated from an analysis of variance between the models, with a small *p*-value indicating a differential dynamic response under treatment. *P*-values were FDR corrected using the R/Bioconductor package qvalue^2^.

## Results

### Morphological Changes Induced by ABA

Studies on *Funaria hygrometrica* (belonging to the Funariaceae as *P. patens*) showed that exogenous application of ABA to protonemal filaments induces the formation of vegetative diaspores (brachycytes or brood cells) and preformed filament breaking points (tmema cells) (Schnepf and Reinhard, 1997). Brachycytes are highly drought tolerant and are still able to grow after 3 years of dormancy (Bopp and Werner, 1993). The number of formed brachycytes depends on the concentration and duration of the ABA application. *Funaria* brachycytes are characterized by thickened cell walls, small vacuoles, plasmatic lipid droplets and reduced plastidal starch (Schnepf and Reinhard, 1997). Tmema cells undergo programmed cell death (PCD) and support fractionation of the filaments so that the brood cells can be propagated (Bopp et al., 1991).

**FIGURE 1:**
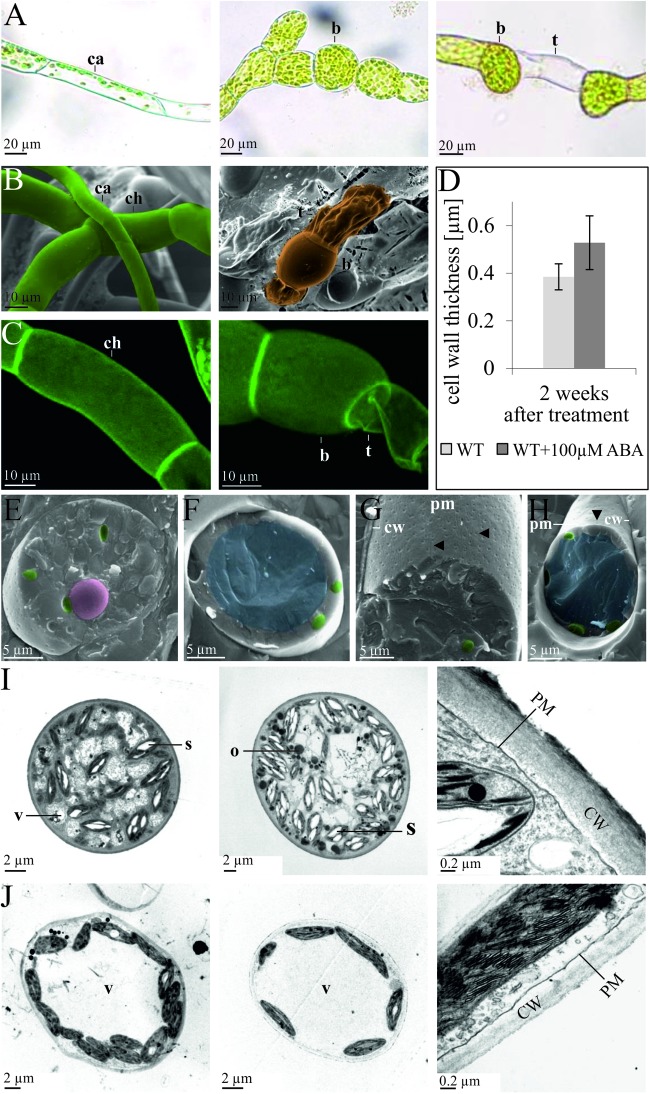
Developmental changes of protonema upon exogenous ABA application. Comparison of protonema before and after ABA application observed via **(A)** light microscopy, **(B)** cryo scanning electron microscopy (SEM), and **(C)** confocal microscopy after propidium iodide staining. The left column shows chloronema (ch) and caulonema (ca) cells prior to ABA application, while the middle and right columns show brachycytes (b, rounded brood cells) and tmema cells (t, preformed filament breaking points) 2 weeks after exogenous application of 100 μm ABA. **(D)** The bar chart shows average cell wall thickness in μm (measured from TEM sections) after 2 weeks of culture without (WT) and with (WT + 100 μm ABA) treatment. Error bars show standard deviation (*n* = 11); the difference is significant (two-sided *t*-test, *p* < 0.01). **(E–H)** Ultrastructural changes upon exogenous ABA application, cryo-SEM. Images are false colored: nucleus (pink), chloroplasts (green), and central vacuole (blue). Cryo-SEM after freeze breaking and etching; **(E,G)** 2 weeks 100 μm ABA, **(F,H)** mock control. In **(E,F)** the cell is broken perpendicular, in **(G,H)** the cell wall (CW) has been removed, allowing to see the plasma membranes’ (PM) outer surface. The central vacuole (blue) clearly visible in **(F,H)** is not recognizable in **(E,G)**. ABA-treated cells exhibit more furrows and a denser patterns of depressions (marked by arrows) that most probably represent negatives (relief) of cellulose synthase complexes. **(I,J)** Ultrastructural changes upon exogenous ABA application, transmission electron microscopy (TEM) images of epon embedded, Osmium-stained cells. **(I)** Five weeks 100 μm ABA, **(J)** without ABA. Intracellular structures are labeled as follows: vacuole (V), starch granules (S), oleosomes (O), plasma membrane (PM), cell wall (CW).

Although the action of ABA on *P. patens* has been considered to be similar to that in *Funaria* (Decker et al., 2006) and also induces brachycytes (Busch et al., 2013), concomitant ultrastructural changes have not been studied in detail. We thus applied ABA to protonemata of *P. patens* in order to analyze whether they respond in a similar fashion as in *Funaria* and to characterize cellular changes. Although the intracellular ABA concentration in *P. patens* is ∼1 μM, increasing to ∼10 μM under abiotic stress (Minami et al., 2003; Beike et al., 2015), ABA is usually applied in the 25–100 μM range in order to achieve developmental changes (Richardt et al., 2010; Busch et al., 2013). Here, we find that exogenous application of 100 μM ABA to protonema leads to the formation of rounded brachycytes and empty tmema cells within 2 weeks (Figure 1A–C). Intracellular changes induced by ABA include the disintegration of the central vacuole, an increase in plastidal starch granules and cytoplasmic oleosomes, and structural changes to the cell wall with an increase in thickness (Figure 1E–H). After 1 week of ABA treatment the cell wall was on average 43% thicker (53 ± 11 μm) than in the control (37 ± 8 μm), which is a significant increase (*t*-test, *p* < 0.01, Figure 1D,I,J). These analyses indicate that brachycytes in *P. patens* form similarly to those in *Funaria*, albeit with the notable difference that more starch granules are formed inside the plastids (Figure 1I,J). *P. patens* brachycytes develop from chloroplast-rich chloronema filaments are characterized by round shape, thick cell wall, loss of the large central vacuole, and increase of starch as well as lipid storage (Figure 1E,I,J). They are thus well suited as vegetative diaspores and represent the end point of the developmental progression triggered by the ABA signaling pathway.

### Identification of Differentially Expressed Genes in Response to ABA

**FIGURE 2:**
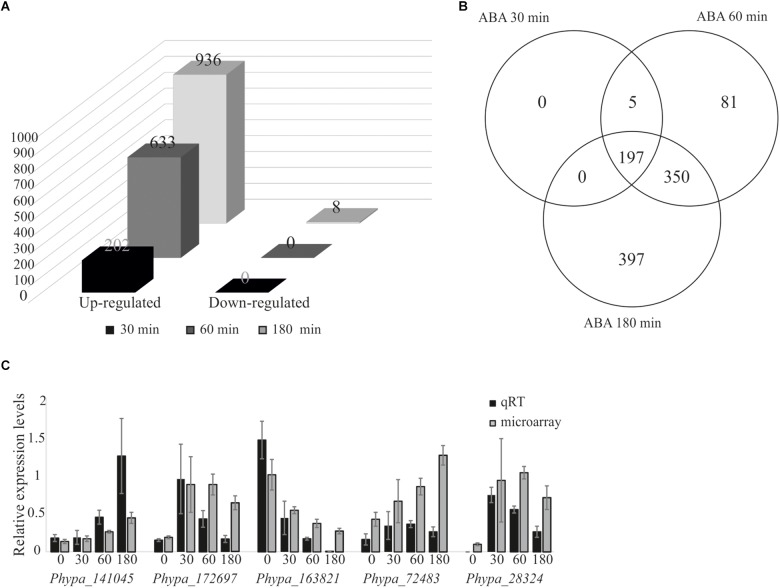
Analysis of ABA-responsive genes and microarray data validation by quantitative real-time PCR. Differentially expressed genes from *P. patens* protonema in response to exogenously applied 10 μM ABA. **(A)** Differentially expressed genes after 30, 60, and 180 min of ABA treatment. **(B)** Venn diagram of ABA-responsive genes after 30, 60, and 180 min of ABA treatment. **(C)** Validation of microarray data by qRT-PCR; expression values were normalized to the reference gene *PpEF1α* (Le Bail et al., 2013). Error bars indicate standard deviation calculated from three biological replicates. The values from the microarray experiments were scaled down by the indicated factors to fit to the qRT-PCR scale: *Phypa_141045, Phypa_172697*, and *Phypa_163821* by 10^4^; *Phypa_72483, Phypa_28324* by 10^3^.

We performed differential gene expression analysis upon ABA application using an established microarray system representing 27,828 out of 35,307 v1.2 genes (Wolf et al., 2010). Protonema grown in liquid culture was exposed to 10 μM ABA, since it was previously shown that 10 μM ABA is sufficient to induce a molecular response but is too low to cause phenotypical changes (Richardt et al., 2010). Transcriptional changes were analyzed at different time points after ABA treatment. We detected a rapid induction of 202 differentially expressed genes (DEGs) after 30 min, 663 genes after 60 min, and 936 genes after 180 min, while no down-regulated genes were detected after 30 and 60 min, and only eight genes were down-regulated after 180 min (Figure 2A and Supplementary Table S1). Five DEGs detected after 30 min were also detected at 60 min but not at 180 min, whereas 197 genes were induced at 30 min and maintained as up-regulated until 180 min of ABA treatment. 81 genes were transiently up-regulated after 60 min of treatment while 397 genes were identified as late ABA-responsive genes, detected only after 180 min of the treatment (Figure 2B). To evaluate the reliability of the microarray results, qRT-PCR was performed on five randomly selected genes that are members of transcription associated genes and represent different expression groups (early and late up-regulation, transient up-regulation, and down-regulation) and which confirmed the microarray data (Figure 2C).

### Cell Wall Related Differentially Expressed Genes

Since we observed cell wall thickening in *P. patens* protonemal cells in response to ABA we searched for genes that might have roles in ABA-mediated cell wall modification. For this purpose, we prepared a list of genes that are either involved in cell wall biosynthesis or related to cell wall regulation from previously published *P. patens* studies. This list contains 71 pectin-related genes (McCarthy et al., 2014), 12 callose synthase genes (Schuette et al., 2009), 11 cellulose synthase genes (Goss et al., 2012), 9 arabinogalactan genes (Fu et al., 2007), 10 cutin synthase genes (Yeats et al., 2014), 30 expansins (Carey and Cosgrove, 2007), and 32 xyloglucan endotransglucosylase/hydrolase genes (Yokoyama et al., 2010; Supplementary Table S2). The list was compared to the identified DEGs and we found that six genes, *Phypa_120256* (galactan galactosyltransferase), *Phypa_207105* (Pectin lyase-like superfamily protein), *Phypa_92683* (glycosyl transferase), *Phypa_114674* (xyloglucan endotransglycosylase), *Phypa_206446* (alpha-expansin), and *Phypa_51702* (arabinogalactan protein) are differentially regulated upon ABA treatment (Table 1). This suggests that the proteins encoded by these genes might be involved in the observed cell wall thickening.

**Table 1:** Cell wall related genes identified to be differentially regulated.

Gene ID	Annotation	Fold changes		
		ABA 30 min	ABA 60 min	ABA 180 min
*Phypa_120256*	Galactan galactosyltransferase	–	2.56	6.41
*Phypa_207105*	Pectin lyase-like superfamily protein	–	3.65	6.45
*Phypa_92683*	Glycosyl transferase	–	2.37	3.11
*Phypa_114674*	Xyloglucan endotransglycosylase	–	–	6.82
*Phypa_206446*	Alpha-expansin	–	–	−5.06
*Phypa_51702*	Arabinogalactan protein	–	–	6.20

*The symbol “−” indicates not significant.*

### ABA Biosynthesis and Signaling Genes

We analyzed whether there is any effect of ABA application on the expression of genes encoding enzymes involved in ABA biosynthesis or proteins acting in ABA signaling. *P. patens* ABA biosynthetic, “core” regulatory components and ABA signaling genes have been identified previously (Stevenson et al., 2016). We calculated fold changes of all genes at 30, 60, and 180 min of ABA application. From this analysis it is obvious that genes encoding enzymes of the ABA biosynthesis pathways are not affected, except two ABA-induced NCED genes that are known to be regulated by ABA in other plant species and encode the enzyme that catalyzes the rate-limiting step of ABA biosynthesis (Sussmilch et al., 2017). Furthermore, almost all genes known to be involved in ABA-dependent signaling and regulation except those encoding the putative ABA receptors and ABI4 were found to be upregulated (Table 2).

**Table 2:** ABA biosynthesis and signaling genes up-regulated with ABA treatments.

		Fold changes		
*Arabidopsis* gene	*P. patens* gene ID	ABA 30 min	ABA 60 min	ABA 180 min
**ABA biosynthetic enzymes**				
ZEP	*Phypa_186228*	–	–	–
SDR	*Phypa_202254*	–	–	–
SDR	*Phypa_125575*	–	–	–
AAO	*Phypa_140802*	–	–	–
AAO	*Phypa_106708*	–	–	–
AAO	*Phypa_172226*	–	–	–
AAO	*Phypa_162514*	–	–	–
MoCo	*Phypa_118134*	–	–	–
NCED	*Phypa_173118*	9.09	8.03	6.34
NCED	*Phypa_159406*	–	–	–
NCED	*Phypa_57876*	–	2.26	4.73
CYP707A	*Phypa_130455*	–	–	–
CYP707A	*Phypa_116547*	–	–	–
CYP707A	*Phypa_69760*	–	–	–
**“Core” regulatory components**				
PYL4-2	*Phypa_209242*	–	–	–
PYL5	*Phypa_213389*	–	–	–
ABI1	*Phypa_32342*	–	4.93	9.40
ABI2	*Phypa_13662*	–	7.99	10.10
OST1-1	*Phypa_194508*	9.01	14.17	21.69
OST1-2	*Phypa_215231*	–	4.99	7.49
OST1-4	*Phypa_106968*	15.23	19.63	20.48
ABI3A	*Phypa_158812*	–	4.22	–
ABI3B	*Phypa_168363*	–	5.15	–
ABI4-like	*Phypa_112999*	–	–	–
**ABA-dependent signaling components**		
HK1-like	*Phypa_124824*	–	3.92	–
PP2C; C subfamily	*Phypa_165686*	2.97	6.42	23
PP2C; C subfamily	*Phypa_232556*	–	7.51	21.2
GRF (14-3-3)	*Phypa_217333*	11.26	32.96	59.83
CBF/NF-Y	*Phypa_8789*	–	7.87	25.84
DREB; subfamily A-2	*Phypa_18841*	–	4.60	4.87
DREB; subfamily A-4	*Phypa_28324*	7.16	9.00	6.26

*The symbol “−” indicates not significant.*

### Functional Enrichment Analysis of ABA Responsive Genes

To examine in which biological process, cellular component and molecular function the ABA responsive genes are involved, GO analysis was performed. Functional categories with FDR corrected *p*-values (*q*-values) < 0.05 are shown in Supplementary Table S3. Enriched terms in “biological process” include response to water deprivation, negative regulation of proteolysis, endopeptidase, peptidase, hydrolase and catalytic activity, monocarboxylic acid metabolic process, lipid metabolic process, and oxidation-reduction process. In the GO “molecular function” category, the most enriched terms are endopeptidase inhibitor activity, enzyme inhibitor activity, oxidoreductase activity and catalytic activity. These results show that plant responses that are required for stress adaptation are enhanced after ABA treatment. In the GO “cellular component” category, the most abundant terms are extracellular space, endoplasmic reticulum, vacuole, organelle membrane, integral and intrinsic component of membrane and cytoplasm. The GO category “protein class” includes protease inhibitor, membrane traffic protein, oxidoreductase, enzyme modulator and transferase. These terms highlight the importance of ABA regulated genes in diverse processes.

### Comparison With Previous Studies

**FIGURE 3:**
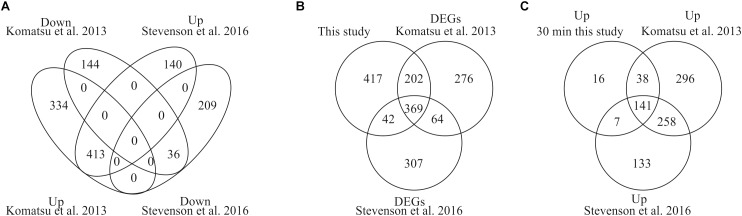
Comparison of DEGs with previously published studies. **(A)** Venn diagram depicting the overlap of DEGs from our study and two previously performed studies by Stevenson et al. (2016) and Komatsu et al. (2013). **(B)** Comparison of all our DEGs with both studies. **(C)** Unique identification of early induced genes by our study.

To provide evidence that our analysis of ABA-induced differential gene expression is robust and novel for the identification of early induced genes we compared our data with two previously published data sets for *P. patens* (Komatsu et al., 2013; Stevenson et al., 2016). Stevenson et al. (2016) performed RNA sequencing analysis using the same ABA concentration as in our study (10 μM) and analyzed transcriptional changes after 60 min of ABA treatment whereas Komatsu et al. (2013) applied a lower ABA concentration of 1 μM and analyzed microarray gene expression after 180 min of ABA treatment. In our study we performed time series analysis of differentially expressed genes in response to 10 μM ABA for 30, 60, and 180 min. The comparison with both data sets indicates that there are 413 commonly up-regulated and 36 commonly down-regulated DEGs (Figure 3A). When we compare all our identified DEGs with both published data sets, we find 571 and 411 commonly regulated DEGs with Komatsu et al. and Stevenson et al., respectively (Figure 3B). Each study detects a set of unique DEGs, with our study detecting the highest number, i.e., 417 (Figure 3B). In addition, we have uniquely identified 16 early ABA inducible genes (Figure 3C and Table 3). Interestingly, seven of those genes encode proteins of unknown function, while five of the annotated genes might be involved in abiotic stress responses, i.e., *Phypa_188559* is an F-box protein family whose homolog is heat-inducible in *A. thaliana* (Lim et al., 2006), *Phypa_51481* and *Phypa_38595* are encoding histidine kinase-related proteins, *Phypa_167873* encodes a chloroplast targeted DnaJ chaperone and *Phypa_90223* a CBS domain protein. These 16 ‘early’ genes were still up-regulated at 60 min of ABA treatment but at 180 min the expression of two genes (*Phypa_117954* and *Phypa_188559*) was decreased, while the remaining 14 genes remained up-regulated (Table 3).

**Table 3:** Uniquely identified early ABA-induced genes.

		Fold changes		
Genes ID	Annotation	ABA 30 min	ABA 60 min	ABA 180 min
*Phypa_127570*	Unknown	5.87	7.62	5.86
*Phypa_138520*	Unknown	7.46	5.79	7.51
*Phypa_163620*	Unknown	18.35	35.34	88.49
*Phypa_167252*	Unknown	4.74	4.40	4.78
*Phypa_224374*	Unknown	9.52	13.85	11.80
*Phypa_75593*	Unknown	30.25	33.24	27.93
*Phypa_92162*	Unknown	5.51	6.14	5.33
*Phypa_167873*	Chaperone protein DNAj chloroplast	7.21	9.09	7.43
*Phypa_223189*	Inositol-tetrakisphosphate 1-kinase 3	3.62	4.87	4.17
*Phypa_38595*	Two-component system sensor kinase	5.15	5.15	5.30
*Phypa_38771*	Chaperone protein	6.45	5.06	4.24
*Phypa_39336*	Copper chaperone	7.77	10.01	18.55
*Phypa_51481*	Multi-sensor hybrid histidine kinase	2.34	2.89	3.32
*Phypa_90223*	CBS domain protein	7.84	10.17	6.99
*Phypa_117954*	Glycerophosphoryl diester phosphodiesterase family protein	4.08	4.04	–
*Phypa_188559*	F-box protein family	5.99	5.51	–

*The symbol “−” indicates not significant.*

### Identification of ABA-Responsive Genes Conserved Between *A. thaliana* and *P. patens*

In order to assess the conservation of ABA-induced changes in gene expression we searched for orthologs of all ABA-regulated *P. patens* genes in the *A. thaliana* genome using the “G:Profiler orthology search” tool (Reimand et al., 2016) with maximum stringency only allowing a single best hit prediction per query gene. Out of a total of 1,030 differentially expressed *P. patens* genes, we found putative orthologs for 621 genes in the *A. thaliana* genome. Some *P. patens* DEGs were found to be paralogs since they were orthologous to the same *A. thaliana* gene leading to a final set of 549 unique *A. thaliana* orthologs of ABA-regulated *P. patens* genes (Supplementary Table S4).

**FIGURE 4:**
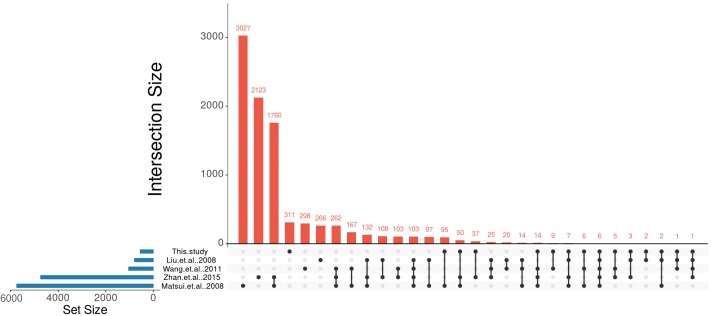
Identification of conserved ABA-regulated genes in *P. patens* and *A. thaliana*. UpSet plot of ABA-responsive genes from four *A. thaliana* studies (Matsui et al., 2008; Wang et al., 2011; Liu et al., 2013; Zhan et al., 2015) and their comparison with putative *A. thaliana* orthologs that are regulated by ABA in *P. patens*.

To compare the ABA-dependent regulation of the identified *A. thaliana* orthologs with the ABA-regulated *P. patens* DEGs we made use of four large-scale studies that analyzed ABA-responsive genes in *A. thaliana* (Matsui et al., 2008; Wang et al., 2011; Liu et al., 2013; Zhan et al., 2015). These studies were selected because they have performed expression analysis with different ABA concentrations, durations of treatment and ages of the plants. In the first selected study 4-weeks-old *A. thaliana* seedlings were treated with 10 μM ABA for 6 h (Liu et al., 2013) and 406 up-regulated and 381 down-regulated genes were detected in response to ABA. In the second study, transcriptional changes were observed from 2-weeks-old *A. thaliana* plants that were subjected to 100 μM ABA treatment for 2 and 10 h (Matsui et al., 2008). In total, this study identified 3,137 up-regulated and 2,619 down-regulated genes. In the third study, 5-weeks-old plants were subjected to 50 μM ABA for 3 h (Wang et al., 2011) and 596 genes were identified as up-regulated and 441 genes were down-regulated. In the fourth study, 2 weeks old plants were subjected to 100 μM ABA for 6 h (Zhan et al., 2015) leading to the identification of 2,380 up-regulated and 2,359 down-regulated genes (Figure 4 and Table 4).

**Table 4:** Comparison of this study with *A. thaliana* studies and conservation analysis.

	Study 1	Study 2	Study 3	Study 4
	Liu et al., 2013	Matsui et al., 2008	Wang et al., 2011	Zhan et al., 2015
Age of *A. thaliana* seedlings	Four-weeks-old	Two-weeks-old	Five-weeks-old	Two-weeks-old
ABA concentration	10 μM	100 μM	50 μM	100 μM
Application duration	6 h	2 and 10 h	3 h	6 h
Up-regulated genes	406	3,137	596	2,380
Down-regulated genes	381	2,619	441	2,359
**Comparison of *A. thaliana* studies**				
Commonly regulated in all four studies			103	
Commonly regulated in studies 2, 3, and 4			262	
Commonly regulated in at least two studies	2,255			
Total non-redundant ABA-regulated genes in *A. thaliana*	8,743			
**Orthologs between *A. thaliana* and *P. patens***				
Total *P. patens* ABA regulated *A. thaliana* orthologs	549			
Commonly regulated orthologs	22	180	42	123
Non-redundant ABA regulated orthologs present in at least one *A. thaliana* study	238			
ABA regulated orthologs detected in all four *A. thaliana* studies	6			

These studies revealed a total number of 8,743 non-redundant ABA-regulated *A. thaliana* genes and their transcriptional overlap inferred from the four studies is depicted in Table 4, Supplementary Table S5, and Figure 4. In total, 103 genes were found to be commonly regulated in all four studies. Liu et al. (2013) had identified the lowest number of genes regulated by ABA. Among the remaining three studies (Matsui et al., 2008; Wang et al., 2011; Zhan et al., 2015) 262 genes are commonly identified to be ABA regulated. In total, 2,255 genes are commonly regulated in at least two studies (Table 4).

Out of 549 unique *A. thaliana* orthologs of ABA-regulated *P. patens* genes, 238 genes were found to be ABA-regulated in at least one of the above mentioned *A. thaliana* studies (Figure 4, Table 4, and Supplementary Table S5). There are six genes (rubber elongation factor protein, protein phosphatase 2C family protein, NAD (P)-binding Rossmann-fold superfamily protein, NON-YELLOWING1, heme oxygenase-like and ABI1) that were found to be differentially expressed in response to ABA in all studies. Among our study and the four *A. thaliana* studies by Matsui et al. (2008), Wang et al. (2011), Liu et al. (2013), and Zhan et al. (2015) are 22, 180, 42, and 123 commonly regulated genes, respectively (Figure 4, Table 4, and Supplementary Table S5). This points to a considerable conservation of ABA-mediated regulation in *P. patens* and *A. thaliana*.

Gene ontology enrichment analysis of the 238 orthologs in which ABA-regulation was conserved in *P. patens* and *A. thaliana* indicates that in the “molecular function” category the majority of genes (110 genes) are involved in catalytic activity, 13 genes have the GO term coenzyme binding and 8 genes are involved in nucleic acid binding. In the “biological process” category the top enrichment GO terms are response to stimulus, response to stress, response to chemical and response to abiotic stimulus. In “cellular component” the highest fold enrichment was observed for GO terms chloroplast membrane, plastid membrane, chloroplast envelope, plastid envelope, vacuolar membrane, whole membrane and vacuole. These terms suggest evolutionary conservation of primary responses in different plastid processes as well as transcriptional regulation by ABA. The complete list of GO-enriched terms is presented in Supplementary Table S3.

In total 621 genes regulated by ABA in *P. patens* are non-conserved between *A. thaliana* and *P. patens* (Supplementary Table S8). Many of those might be lineage specific, since only 117 of these have GO terms associated with them in the most recent gene annotation version (Lang et al., 2018). There are 311 *P. patens* DEGs that have putative orthologs in *A. thaliana* but are not ABA regulated in *A. thaliana*, while there are 310 genes that are both conserved and regulated by ABA in both organisms. The ABA regulated *P. patens* genes that are conserved with *A. thaliana* do not show a dominant over-representation of GO terms (Supplementary Figure S1), regardless of whether the genes are also regulated by ABA in *A. thaliana* or not. However, many metabolic processes enabled by conserved genes are halted upon ABA action, as can be seen from many depleted terms that are associated with genes controlling macromolecule metabolism. The 409 ABA regulated *P. patens* genes not conserved with *A. thaliana* do not show many dominant depletion terms, but many enriched terms that reflect responses to abiotic stimuli/stresses, among them UV, cold and ROS (Supplementary Figure S1).

### Time Series Analysis

**FIGURE 5:**
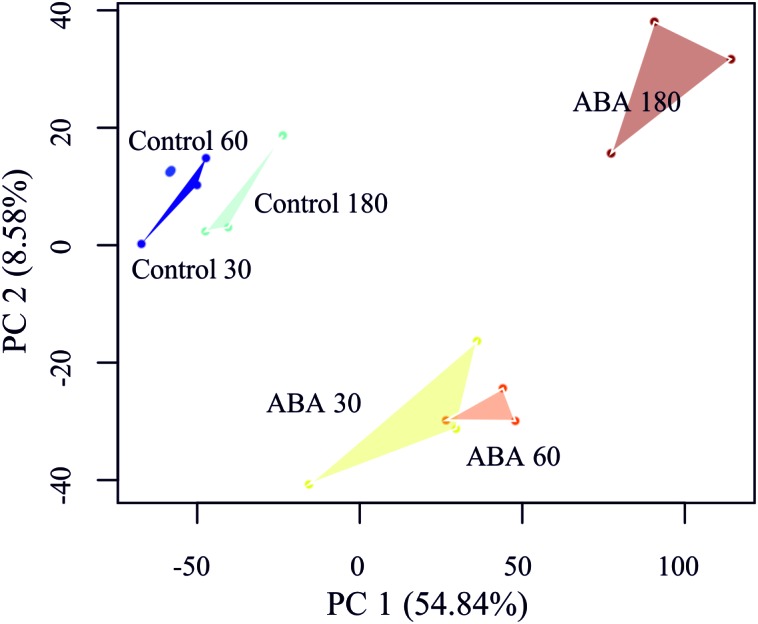
Principal component analysis (PCA) of the *P. patens* transcriptome time series. Replicates have been linked by a convex hull to visualize the sample distances.

**FIGURE 6:**
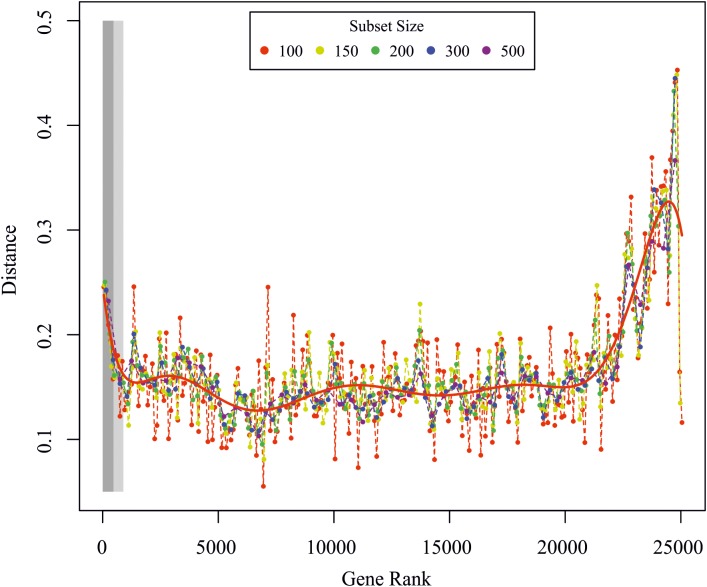
Global transcriptome analysis. Euclidean distance between the global and ordered gene subset response for subsets of different sizes. The red line is a polynomial fit to all data points. The light and dark rectangles depict the significantly regulated genes from the *F*-test for the FDR corrected *p*-values < 0.01 and <0.05, respectively. Genes were ranked according to the *p*-value from the *F*-test analysis.

In order to detect DEGs under ABA stimulation over time, we measured the transcriptome response at 30, 60, and 180 min after ABA stimulation. To learn which genes control the developmental decision toward brachycytes, we performed a global transcriptome time course analysis as in Busch et al. (2013). A principal component analysis (PCA) on the transcriptomes shows a clear separation along the first principal component (PC 1) of the ABA-stimulated gene response vs. the control time points (Figure 5). Furthermore, there is a transient gene response at the 30 and 60 min time points of ABA treatment, which are separated along the second principal component (PC 2). Next, we analyzed which genes respond differently over time in the ABA-stimulated vs. the control cells by fitting a 3rd order polynomial to response of each gene under treatment and control separately and together and comparing the goodness of fit via analysis of variance (ANOVA) (Supplementary Table S6 and Figure 6). Using this analysis, we found 1,792 DEGs regulated over time at a FDR adjusted *p*-value threshold < 0.001 (as compared to 1,030 in the pairwise analyses). Among these there are 63 out of 1,380 annotated genes that encode transcription-associated proteins (TAPs). The analysis confirms the significant differential regulation of the five TAPs tested (Figure 2C), Phypa_141045, Phypa_172697, Phypa_163821, Phypa_72483, and Phypa_28324 (Supplementary Figure S2).

### Crosstalk

**FIGURE 7:**
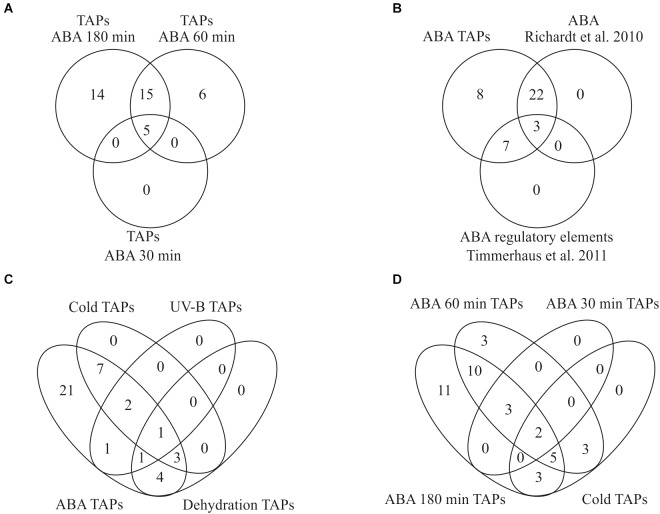
Overlap of transcription associated proteins (TAPs) in our and other stress related studies. **(A)** Identification of differentially expressed TAPs after 100 μM ABA application for 30, 60, and 180 min. **(B)** Overlap of ABA-regulated TAPs found in this study with Richardt et al. (2010) and Timmerhaus et al. (2011) **(C)** All TAPs differentially expressed during cold stress, dehydration and UV-B light are also differentially regulated in response to ABA. **(D)** Comparison of differentially expressed genes in response to cold and ABA.

In total, 40 differentially expressed TAPs were found in all pairwise comparisons and 35 of these were also detected as differentially expressed according to the time series analysis (Supplementary Table S7). Only one TAP encoding a member of the AP2/EREBP TF family was down-regulated (*Phypa_163821*) whereas the other 39 TAPs were up-regulated in response to ABA. Most of the differentially expressed TAPs belong to the group of transcription factors (TF, 35) whereas only a few are transcriptional regulators (TR, 5). The latter are members of the Argonaute family mainly involved in small RNA-directed regulation of nuclear-encoded transcripts (*Phypa_172642* and *Phypa_141045*), or they belong to the Sigma 70-like protein family that act as cofactors of the plastid-encoded RNA polymerase to control plastidic gene expression (*Phypa_86427*, *Phypa_160930*, and *Phypa_230140*). The five differentially expressed TAPs that were up-regulated already after 30 min stayed activated after 60 and 180 min. Half of the differentially expressed TAPs were up-regulated after 60 min and remain up-regulated even after 180 min (Figure 7A).

Out of 1,792 genes that were differentially regulated over time according to our time series analysis, 328 genes were classified as ABA-responsive according to the criteria defined by Timmerhaus et al. (2011). Out of the 40 differentially expressed TAPs from the pairwise comparisons an ABA responsive element was predicted for ten of the differentially expressed TAP genes (Timmerhaus et al., 2011) and an activation by ABA or salt was shown for 25 using a TAP-specific microarray (Richardt et al., 2010). The list of ABA-regulated TAPs found in our study thus fully incorporates both sets indicating the high sensitivity and reliability of this study (Figure 7B).

Since ABA plays an important role in the stress response of plants, we compared the set of differentially expressed TAPs identified in our analysis to other studies in *P. patens* that analyzed transcriptional changes in response to different abiotic stresses. Strikingly, we found that all TAPs that were previously identified to be differentially expressed during cold stress, dehydration and UV-B radiation are also differentially regulated in response to exogenously applied ABA (Figure 7C), supporting a common role of ABA in diverse molecular stress responses. We found 13 TAPs activated by ABA and cold stress (Beike et al., 2015), five activated by ABA and UV-B (Wolf et al., 2010) and nine activated by ABA and dehydration (Hiss et al., 2014). A single TAP gene (*Phypa_126548*) that is activated by ABA and all other stresses encodes an AP2/EREBP family protein previously shown to be prominently involved in the *P. patens* stress response (Richardt et al., 2010; Hiss et al., 2014). The cold-responsive TAPs that were also identified in our data set show an activation by cold primarily after 8–24 h. In our ABA time course most of them show an activation at the two later time points (60 and 180 min), suggesting that their activation upon cold requires ABA biosynthesis and/or ABA-release from glucosyl esters (Figure 7D).

## Discussion

The first land plants had to evolve processes that are required for successful land colonization such as drought and freezing tolerance (Kenrick and Crane, 1997; Charron and Quatrano, 2009; Rensing, 2018; Reski, 2018). Many of these features are present in extant land plants and hence are evolutionarily conserved since the split of mosses and the vascular plant lineage. The moss *P. patens* is highly tolerant against dehydration, salt and osmotic stress (Frank et al., 2005b) and cells from the filamentous protonemal tissue can undergo differentiation into vegetative diaspores (brachycytes) under unfavorable growth conditions such as extended periods of dehydration. Furthermore, desiccation and osmotic stress result in the accumulation of ABA (Minami et al., 2005; Xiao et al., 2018), and brachycyte formation can be induced by exogenous ABA application (Decker et al., 2006), indicating that elevated ABA levels are sufficient to provoke all transcriptional changes underlying this specific developmental fate. ABA application results in the development of brachycytes that are characterized by thickened cell walls, tiny vacuoles and the storage of lipids and starch (Figure 1). The formation of brachycytes is ABA-dependent as ABA non-responsive mutants (Ppanr) do not produce any brachycytes (Minami et al., 2006; Saruhashi et al., 2015; Stevenson et al., 2016).

Cold stress causes an inhibition of protonemal growth and provokes the formation of chlorotic and roundish cells that resemble brachycytes. In addition, gene expression analyses from cold treated *P. patens* samples provided evidence for an involvement of ABA during cold responses (Beike et al., 2015). In our study we also detect a comprehensive overlap between cold and ABA mediated gene regulation since we found all previously identified cold regulated TAPs to be regulated in response to exogenously ABA application.

Triggering brachycyte formation by exogenous ABA depends on the applied ABA concentration, with 10 μM ABA few brachycytes were observed whereas 100 μM ABA caused the formation of a large number of brachycytes. The formation of empty tmema cells next to the brachytes, possibly by PCD, causes the disruption of protonema filaments which supports the release and propagation of brachycytes. The outgrowth of the vegetatively formed brachycytes resembles the germination process of spores. When brachycyte growth is initiated, the cell wall ruptures and the outgrowth is covered by a newly formed cell wall as in germinating spores (Schnepf and Reinhard, 1997).

Interestingly, the developmental decision to form brachycytes is already made after 180 min of ABA application. Replacement of the ABA-containing medium after 180 min resulted in formation of brachycytes, although less than in the control experiment in which the medium was not exchanged (Supplementary Table S9).

In order to look for commonalities in the developmental process leading to formation of spores and brachycytes we compared the transcriptome of protonemata after 3 h of ABA treatment with the transcriptome of brown sporophytes (Hiss et al., 2014), a developmental stage consisting primarily of postmeiotic cells that are determined to develop into spores. The GO terms associated with the genes having their highest expression in either of the two conditions are quite different (Supplementary Figure S3), there is no overlap of terms between the two conditions. Hence, the developmental route leading to the two forms of diaspores seem to differ significantly. Interestingly, the GO word cloud describing the highly expressed genes unique to brachycyte formation very much resemble those of the ABA regulated genes conserved between *P. patens* and *A. thaliana* (Supplementary Figures S1, S3).

In *P. patens* ABA treatment results in widespread changes including changes in the gene expression related to cell wall modifications. It was previously hypothesized that differential expression of proteins like pectin methylesterase, proline-rich cell wall proteins and leucine rich repeat domain proteins are probably involved in cell wall modifications (Tintelnot, 2006; Cuming et al., 2007). Similarly, in the study of Schipper et al. (2002) it was shown that expansin genes that are associated with cell wall modifications are up-regulated by ABA in a concentration-dependent manner. We also identified several differentially regulated ABA-responsive genes that encode proteins related to cell wall modifications such as galactan galactosyltransferase, pectin lyase-like superfamily protein, glycosyl transferase, xyloglucan endotransglycosylase, alpha expansin and arabinogalactan protein. The observed ABA-induced differential expression of genes encoding cell wall modifying enzymes is consistent with the ABA-mediated morphological changes, in particular the formation of thick walled brachycytes and tmema cells with drastically reduced cell walls.

The formation of tmema cells that usually flank the brachycytes is probably achieved by PCD as suggested earlier (Goode et al., 1993a; Decker et al., 2006). Indeed, after 180 min we could observe an ABA-responsive induction of genes that can be considered to play a role in tmema cell formation. These include a gene encoding a metacaspase (MC9, *Phypa_140308*, 11.25 fold increase), two autophagy-related genes (*Phypa_8878*: 6.33 fold up-regulation and *Phypa_223103*: 3.76 fold up-regulation) and a senescence-associated gene coding for a triacylglycerol lipase-like protein (SAG, *Phypa_161303*, 16.3 fold up-regulation). The metacaspase MC9 is one of five evolutionary conserved core developmental PCD marker genes in the green plant lineage (Olvera-Carrillo et al., 2015). Autophagy is an intracellular destructive mechanism to degrade intracellular proteins, metabolites and organelles for recycling and is further required for developmental PCD (Minina et al., 2014; Kurusu and Kuchitsu, 2017) and SAG can similarly play a role in the degradation of cells (Muhlenbock et al., 2008; Wituszynska et al., 2013). The relatively low number of PCD-related DEGs, and the fact that GO terms like cell death and PCD are not enriched, suggest that the developmental triggering of brachycyte formation preceded tmema formation.

Many genes coding for core regulatory components of the ABA signaling pathway were induced by increasing ABA concentrations, whereas genes encoding enzymes for ABA biosynthesis remain unchanged, with the exception of members of the NCED gene family that are considered to catalyze the rate-limiting step in stress-provoked ABA biosynthesis (Frey et al., 2012; Behnam et al., 2013). In the study of Stevenson et al. (2016) genes encoding NCEDs and abscisic aldehyde oxidase (AAO) were shown to be up-regulated by ABA, but we only detected up-regulation of NCED genes. However, we observed the up-regulation of a large number of genes that encode for core components of the ABA signaling pathway including *ABI1, ABI2, OST1-1, OST1-2, OST1-4, ABI3A*, and *ABI3B* that coincides with Stevenson et al. (2016). Similarly, the up-regulated ABA dependent signaling component genes in both studies are *HK1, PP2C; C subfamily* (both members)*, GRF (14-3-3), CBF/NF-Y, DREB; subfamily A-2 and DREB; subfamily A-4*. In the case of the moss *P. patens* ABA is obviously involved in controlling the formation of vegetative diaspores that allow the plants to survive under unfavorable environmental conditions. We conclude that the ABA biosynthesis and signaling pathway is an ancestral land plant feature, which controls a large range of biological processes including the adaptation to abiotic stress, mainly dehydration, osmotic stress and cold (Minami et al., 2003; Khandelwal et al., 2010; Stevenson et al., 2016). Furthermore, increased ABA levels in moss are required for the formation of vegetative spores and in seed plants for stomatal control and seed dormancy.

Osmoregulation seems to be a major process mediated by ABA. In *P. patens* genes encoding for alpha/beta amylases, sugar transporters, dehydrins, osmosensor histidine kinases and early-responsive to dehydration stress proteins are induced by ABA with a most probable function in osmoregulation (Cuming et al., 2007; Arif et al., 2018). Group I LEA proteins can be activated in angiosperms by exogenously applied ABA (Kamisugi and Cuming, 2005) and are involved in the regulatory period of dehydration at the end of seed development. Up-regulation of several *LEA* genes upon ABA application has been shown by Kamisugi and Cuming (2005), which we could also confirm. Here we report eight additional ABA regulated *LEA* genes not previously shown to be regulated by ABA (*Pp1s233_54V6.1, Pp1s235_65V6.1, Pp1s52_212V6.1, Pp1s379_42V6.1, Pp1s60_54V6.1, Pp1s54_73V6.1, Pp1s13_112V6.1*, and *Pp1s33_47V6.1*). The first four genes were described as LEA proteins by Artur et al. (2018) using Pfam domain predictions: *Pp1s233_54V6.1* and *Pp1s235_65V6.1* belong to LEA2 family while *Pp1s52_212V6.1* and *Pp1s379_42V6.1* are LEA4 family members. In the latest version (v3.3) of the *P. patens* gene annotation (Lang et al., 2018), the remaining four genes were annotated as LEA proteins. This provides evidence for common ABA-mediated molecular processes during diaspore development in angiosperms (seeds) and mosses (brachycytes).

We found considerable conservation of ABA-mediated responses between *P. patens* and *A. thaliana*. In total, 621 out of 1,030 ABA-regulated genes in *P. patens* have putative orthologs in *A. thaliana*. Most of them are known in *A. thaliana* to be associated with stress adaptation, including genes involved in calcium binding, low temperature and salt response, or coding for heat shock proteins, oxidoreductases, molecular chaperons, LEA proteins, stress-related transcription factors, ABA signaling proteins and sugar metabolism enzymes.

Abscisic acid is activated by stress conditions in several plant species (Nakashima and Yamaguchi-Shinozaki, 2013; Vishwakarma et al., 2017). We show here that TAP genes that are differentially regulated in *P. patens* during stress conditions like drought, UV-B light and cold are activated by ABA treatment as well. Nine of these differentially expressed TAP genes belong to the AP2/EREBP TF family. Members of this family control developmental processes as well as stress acclimation responses (Dietz et al., 2010).

The temporal analysis of the transcriptome finds nearly twice as many genes putatively involved in carrying out ABA-dependent development as the pairwise DEG analyses. Of the 176 cell wall related genes (Supplementary Table S2), 66 are found to be regulated in the time course analyses, supporting the validity of the analysis and the impact of ABA on cell wall restructuring.

Among the time course DEGs are 63 TAPs that might be important for regulating the ABA-induced developmental progression to vegetative diaspores. Most TAPs are up-regulated at 30 or 60 min and stay up-regulated (Supplementary Table S7). Interestingly, the TAPs activated upon ABA treatment incorporate all TAPs previously predicted to be ABA responsive (Timmerhaus et al., 2011) or shown to be ABA-regulated (Richardt et al., 2010; Figure 7B). Moreover, all TAPs previously found to be regulated by other stresses (UV-B, cold, dehydration) are also induced by ABA (Figure 7C). This suggests that the developmental progression yielding brood cells can be triggered by different stresses and is controlled by ABA.

## Data Availability

This manuscript contains previously unpublished data. The microarray data reported in this manuscript were uploaded to ArrayExpress under the ID: E-MTAB-5492.

## Author Contributions

MT performed the microarray and qRT-PCR experiments. MA, MH, RM, and HB analyzed the data. ST analyzed the developmental changes of protonema upon exogenous ABA application and performed electron microscopy. MA wrote the manuscript with the help of MH, HB, SR, and WF. RR, SR, and WF designed the experiments.

## Conflict of Interest Statement

The authors declare that the research was conducted in the absence of any commercial or financial relationships that could be construed as a potential conflict of interest.

## Footnotes

1 http://www.wordle.net/

2 http://github.com/jdstorey/qvalue

## Supplementary Material

FIGURE S1 GO bias analysis (biological process) for conserved ABA regulated **(A)**, conserved, but not ABA regulated **(B)** and non-conserved genes **(C)** between *P. patens* and *A. thaliana*. Green font color marks over-represented, red font color marks under-represented GO terms, and darker colors indicate a lower *q*-value.

FIGURE S2 Bar charts of 40 differentially expressed *P. patens* TAPs in the pairwise comparisons of control and all three ABA treated time points.

FIGURE S3 GO bias analysis of the genes expressed most highly in protonemata treated for 3 h with ABA (250, **A**), and in brown sporophytes (55, **B**). Colors as in Supplementary Figure S1.

TABLE S1 *P. patens* differentially expressed genes with the ABA treatment.

TABLE S2 *P. patens* cell wall related genes.

TABLE S3 Gene ontology enrichment analysis of all DEGs in *P. patens* and 238 conserved ABA-regulated genes between *P. patens* and *A. thaliana*.

TABLE S4 ABA responsive genes conserved between *A. thaliana* and *P. patens.*

TABLE S5 Commonly ABA regulated genes between *P. patens* and *A. thaliana* studies.

TABLE S6 Ranked list of differentially regulated genes according to the *F*-test for differential gene regulation between ABA stimulated samples and controls.

TABLE S7 Genes identified as regulated by different treatments in different studies.

TABLE S8 Conservation/non-conservation analysis of ABA regulated genes between *P. patens* and *A. thaliana*.

TABLE S9 Numbers of brachycytes formed after 2 weeks of constant vs. 180 min ABA application.
