# Supplementary figures and images for: ABA-Induced Vegetative Diaspore Formation in Physcomitrella patens
Source: Front Plant Sci. 2019 Mar 19;10:315. doi: 10.3389/fpls.2019.00315 (PMC6433873; doi:10.3389/fpls.2019.00315)

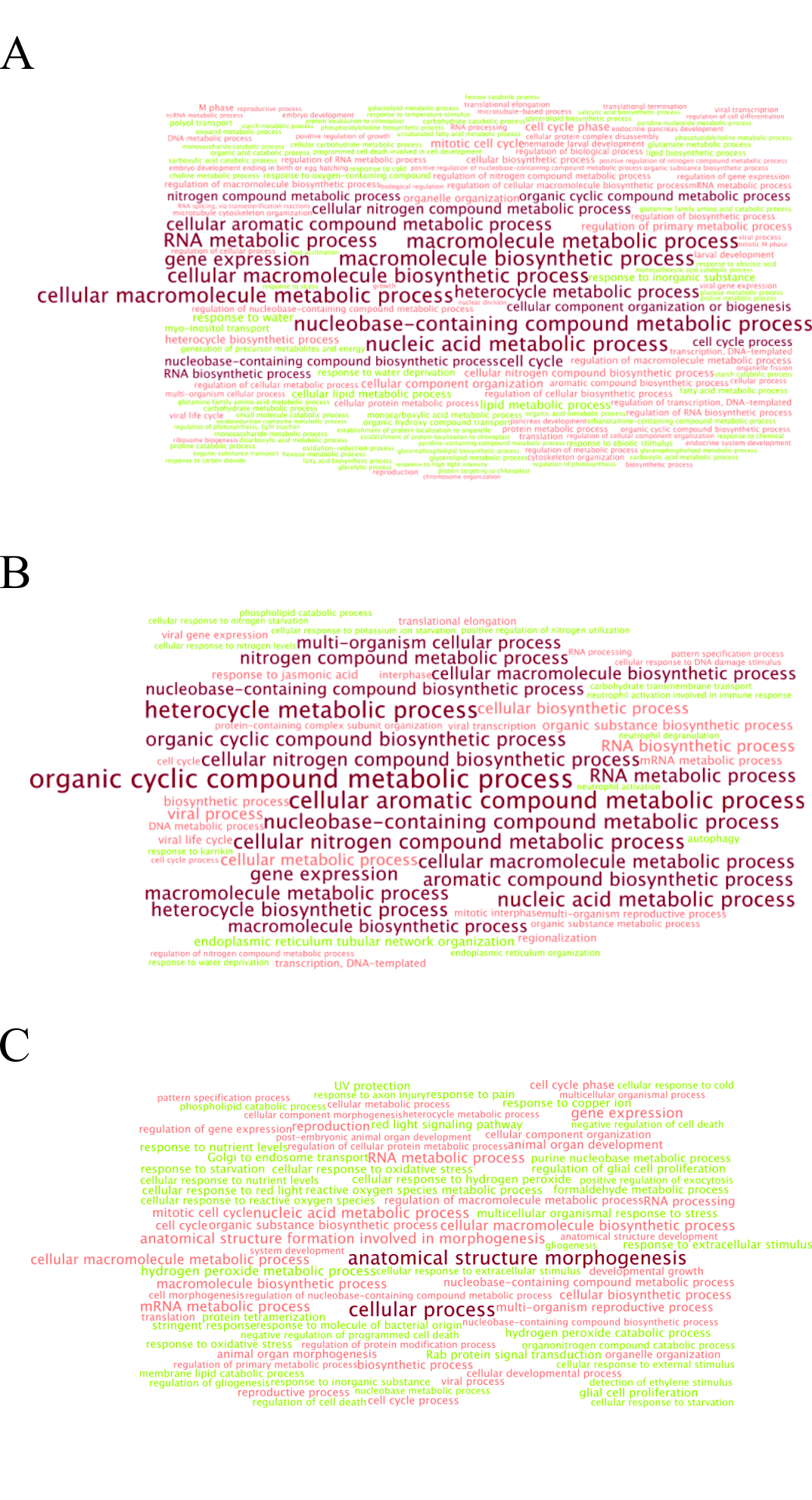

Supplement: FIGURE S1 — GO bias analysis (biological process) for conserved ABA regulated (A), conserved, but not ABA regulated (B) and non-conserved genes (C) between P. patens and A. thaliana. Green font color marks over-represented, red font color marks under-represented GO terms, and darker colors indicate a lower q-value. [file Image_1.JPEG]

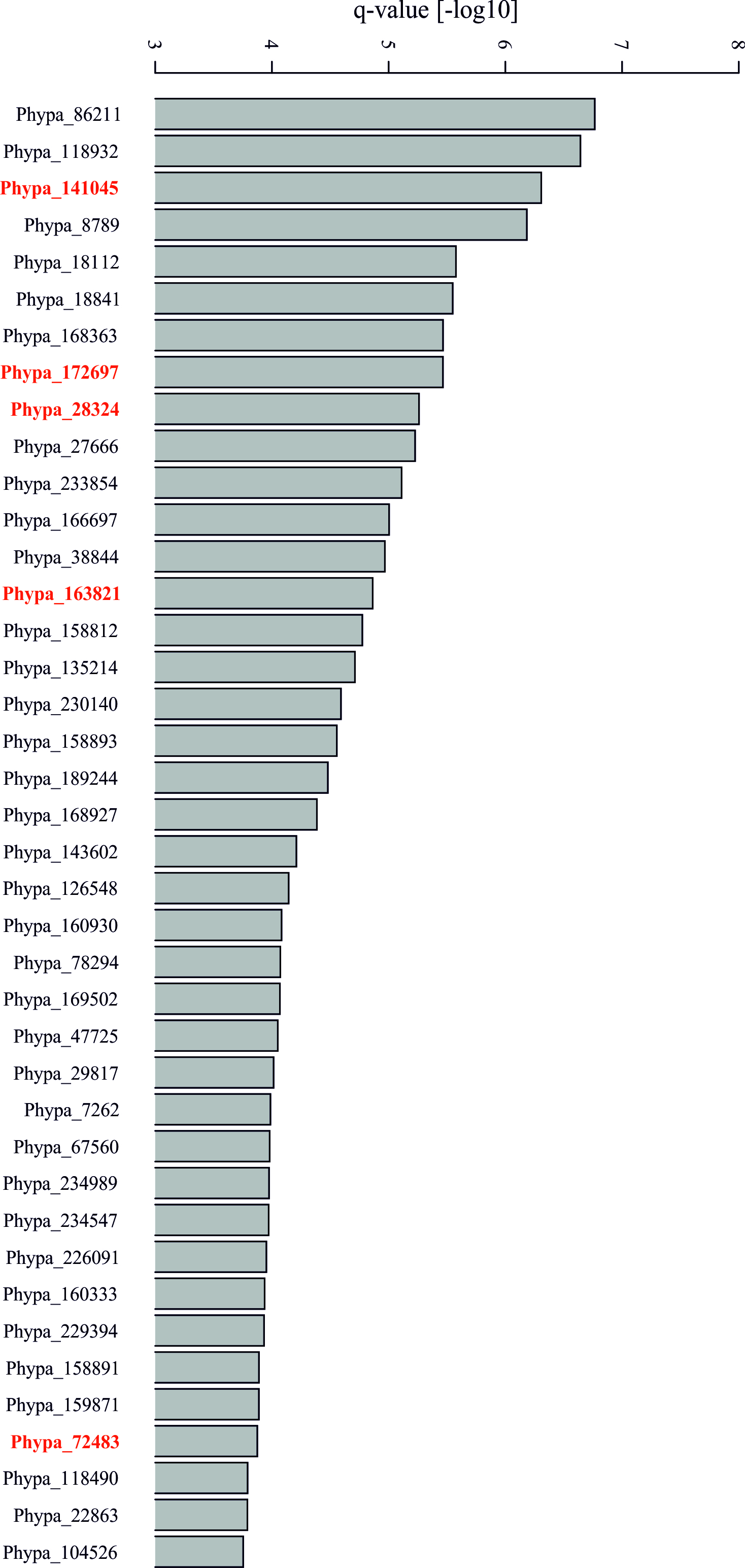

Supplement: FIGURE S2 — Bar charts of 40 differentially expressed P. patens TAPs in the pairwise comparisons of control and all three ABA treated time points. [file Image_2.JPEG]

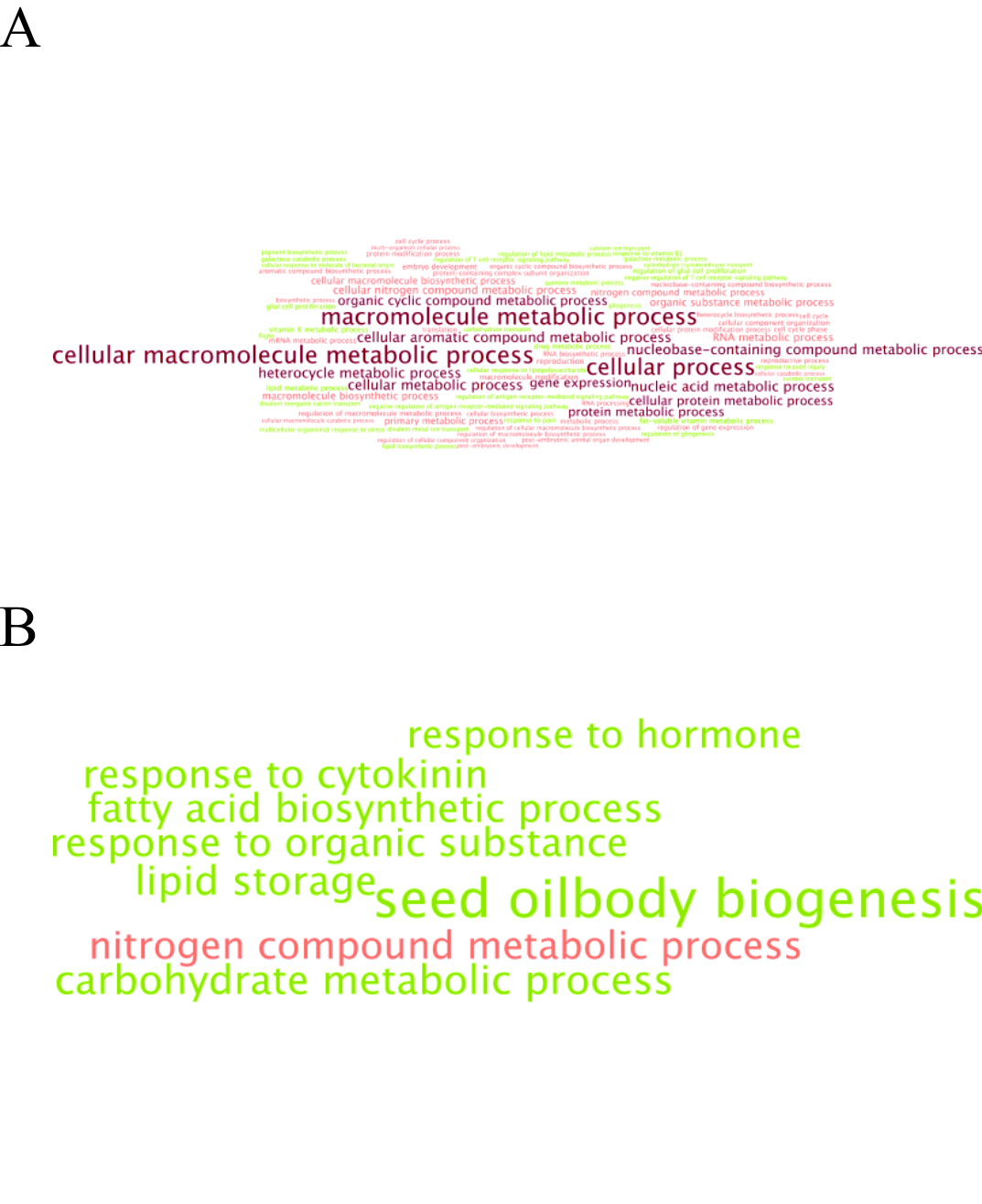

Supplement: FIGURE S3 — GO bias analysis of the genes expressed most highly in protonemata treated for 3 h with ABA (250, A), and in brown sporophytes (55, B). Colors as in Supplementary Figure S1. [file Image_3.JPEG]
